# A New Functional Wheat Flour Flatbread (Bazlama) Enriched with High-β-Glucan Hull-Less Barley Flour

**DOI:** 10.3390/foods13020326

**Published:** 2024-01-20

**Authors:** Hamit Koksel, Zeynep Hazal Tekin-Cakmak, Sena Oruc, Gozde Kilic, Kubra Ozkan, Buket Cetiner, Osman Sagdic, Francesco Sestili, Abderrazek Jilal

**Affiliations:** 1 Department of Nutrition and Dietetics, Health Sciences Faculty, Istinye University, İstanbul 34010, Türkiye; dytgozdekilic@gmail.com (G.K.); kubraozkan1907@gmail.com (K.O.); 2 Department of Food Engineering, Faculty of Chemical and Metallurgical Engineering, Davutpasa Campus, Yildiz Technical University, Istanbul 34349, Türkiye; hazal.cakmak@yildiz.edu.tr (Z.H.T.-C.); osagdic@yildiz.edu.tr (O.S.); 3 Department of Nutrition and Dietetics, Faculty of Health Sciences, Acibadem University, İstanbul 34752, Türkiye; sena.oruc@acibadem.edu.tr; 4 Department of Quality and Technology, Field Crops Central Research Institute, Ankara 06170, Türkiye; buket.cetiner@tarimorman.gov.tr; 5 Department of Agriculture and Forest Sciences (DAFNE), University of Tuscia, 01100 Viterbo, Italy; 6 National Institute for Agricultural Research Morocco (INRAM), Rabat P.O. Box 415, Morocco; abderrazek.jilal@inra.ma

**Keywords:** barley flour, bazlama, antioxidant capacity, glycemic index, mineral compositions

## Abstract

Although the Med-Diet is a healthy diet model, it is affected by current dietary habits. Therefore, new foods with improved nutritional value should be developed to respond to the needs of people following the Med-Diet. This study was focused on developing high-β-glucan flat bread (bazlama) with a relatively lower GI. A bread wheat (*cv*. Tosunbey) flour was enriched with the flour of a high-β-glucan-content hull-less barley (*cv*. Chifaa) flour (15, 30, 45 and 60%) to develop a functional bazlama. The nutritional and technological properties of bazlama samples enriched with barley flour were compared with the ones produced from bread wheat. All of the barley flour-enriched bazlama samples had higher yellowness values (b*) than the control (both crumb and crust), which is generally preferred by the consumers. Texture results indicated that bazlama samples became harder with the increase in barley flour supplementation level. The results showed that 3 g of β-glucan can be provided from the barley flour-enriched bazlama samples (at 45 and 60% levels), and this is the limit to carry health claims. The bazlama samples enriched with barley flour were richer in Mg, K, Mn, Fe, and Zn minerals than the control (100% Tosunbey flour). While the glycemic index (GI) of commercial bread wheat and Tosunbey bazlama samples were high (88.60% and 79.20%, respectively), GI values of the bazlama samples enriched with 60% (64.73) and 45% barley flour (68.65) were medium. The lower GI values of barley flour-enriched bazlama samples are probably due to the higher β-glucan contents of the bazlama samples. Additionally, as the barley flour supplementation level of the bazlama samples increased, the phenolics and antioxidant capacities of free and bound extracts increased compared to bread wheat bazlama. The results indicated that hull-less barley (*cv*. Chifaa) with high β-glucan content may be utilized at relatively higher levels (45 and 60%) to produce bazlama with improved nutritional properties.

## 1. Introduction

A wide range of breads have been produced and consumed around the world over the centuries. It is still the main staple for a significant number of people around the world. Bread has a special role in meeting carbohydrate, plant-based protein, B group vitamin, mineral, and daily energy requirements in human nutrition [1]. It is claimed that 20–50% of people’s daily energy needs are met by bread [2]. Bread has a good potential to develop a functional food due to its structural suitability and is a staple food with a high consumption level [3]. The majority of consumption consists of bread manufactured from refined bread wheat flour [4]. There is an urgent need to increase the consumption of whole grain and mixed grain breads to improve the health benefits.

The glycemic index (GI) of barley (34–70) is usually lower than other cereals (46–80 for corn, 55–85 for rice, and 52–75 for wheat), but GI is also affected by the way the food is processed or consumption in combination with other foods. The plasma glucose-lowering effect of barley-containing foods may be attributed to their β-glucan content and amylose/amylopectin ratio [5]. Compared to bread wheat, barley is rich in protein, dietary fiber, especially B group vitamins, and minerals. Barley is called a functional grain because it contains β-glucan, soluble dietary fiber, and phytochemicals [6]. In terms of phenolic components, barley grain contains ferulic acid (68%) as the main component and small amounts of various other bioactive compounds, including 2,4-dihydroxybenzoic acid, syringic acid, p-hydroxybenzoic acid, sinapic acid, vanillic acid, and coumaric acid. It is also rich in flavanols, anthocyanins, and proanthocyanidins [7].

Barley is mainly used for animal feeding and malting. It is estimated that around 2% of barley grain is used in food production. Due to its health-beneficial effects, consumption of barley in human nutrition has been increasing in recent years [7]. About 65–68% carbohydrate, 10–17% protein, 4–9% β-glucan, 2–3% free lipids, and 1.5–2.5% minerals are found in barley grain [8]. It has been proven that a β-glucan-rich diet can improve immune system function and protect against hypertension, stroke, cardiovascular disease, and Type 2 diabetes [5]. In a study organized with 44 men with elevated serum cholesterol levels who consumed 7 g of β-glucan per day, it was found that visceral obesity was recruited, and LDL and total serum cholesterol and total cholesterol levels were reduced [9]. 

The positive health effects of β-glucan can be summarized as anti-inflammatory, anti-carcinogenic, antioxidant, and immunomodulatory and being able to improve the functionality of gastrointestinal and cardiovascular systems [10]. β-glucans are reported to have cardiovascular protective activity, anti-diabetic properties, and an improving effect on microbiota health [11,12,13,14,15,16]. The immunostimulatory activity of β-glucan is due to its capacity to bind to specific membrane receptors on immunologically competent cells, including monocytes, neutrophils, macrophages, natural killer cells, and dendritic cells. The immunostimulatory activity of β-glucan is based on the macrophage activation mechanism. Due to the presence of specific receptors (e.g., CR3, TLR-2, Dectin-1), macrophages recognize the β-glucan structure that initiates a cascade of both cellular and humoral immune responses [17].

The health claim for barley was approved in 2006 by the U.S. Food and Drug Administration (FDA) based on studies demonstrating that frequent consumption of barley products can prevent or treat cardiovascular illnesses by reducing plasma cholesterol. Barley’s health-related functions are mainly attributed to its high β-glucan and tocopherol content. In order to bear the claim on food labeling: “Foods should provide at least 3 g/day of β-glucans from barley, barley bran, or mixtures of non-processed or minimally processed barley β-glucans in one or more servings. A minimum of 0.75 g of β-glucans per serving is recommended, or one-fourth of the 3 g daily amount specified above, to assist consumers to choose foods to suit their diet” [18]. In most Mediterranean countries, the average per capita consumption of bread and/or other cereal products (couscous, bulgur, pasta, cereal soups) is around 200 g/day. Hence, there is a good possibility of consuming 3 g of β-glucans per day if barley is included in the diet of Mediterranean people in different forms. Chifaa is a specific cultivar of barley, which is a rich source of β-glucan, phenolic acids, and other phytochemicals [19]. Hence, it has the potential to be used in developing healthy food products.

It has been reported in various studies that contemporary diets have been causing serious consequences in human health, raising the incidence of non-communicable diseases such as cardiovascular diseases, cancer, type 2 diabetes, and obesity. The traditional Med-Diet is a healthy diet model; however, it is also affected by the changes in current dietary habits. Therefore, there is an important need to develop new Med-Diet foods with improved nutritional value to respond to the needs of the Mediterranean populations as well as others following the Med-Diet. Hence, this study was focused on developing high-β-glucan and lower-GI flat bread (bazlama), a traditional food in the Mediterranean area. This study aimed to investigate the effects of supplementing wheat flour with high-β-glucan hull-less barley (*cv*. Chifaa) flour on composition, quality characteristics, nutritional attributes, and potential health benefits of a flatbread (bazlama). The focus was on quality evaluation of bazlama samples produced from hull-less barley flour-enriched (15, 30, 45, and 60%) wheat flour. The changes in the β-glucan contents, in vitro glycemic index values, phenolic contents, antioxidant capacities, and mineral compositions of barley flour-enriched bazlama samples were also evaluated. By systematically examining these parameters, a deeper understanding of the effects of integrating high-β-glucan hull-less barley flour into bazlama is expected to be achieved for developing novel, nutritionally enriched products for health-conscious consumers.

## 2. Materials and Methods

### 2.1. Materials

Bread wheat (*cv.* Tosunbey, *Triticum aestivum* L.) and barley (*cv.* Chifaa, *Hordeum vulgare* L.) were used as the raw materials for bazlama production. The barley *cv*. Chifaa is a high-β-glucan hull-less barley produced in Marchouch, Morocco, in the 2021–2022 growing season. The bread wheat variety (*cv*. Tosunbey) is a hard white winter wheat with strong gluten properties obtained from Turkish Grain Board, Ankara, Turkey. Commercial flour (Sinangil, Eksim milling Co., İstanbul, Türkiye), salt, sugar, and dry yeast were bought from local markets in İstanbul, Turkey. All of the solvents and reagents used in this study were purchased from Sigma-Aldrich (St. Louis, MO, USA). Glucose and beta-glucan assay kits were from Megazyme (Megazyme International, Wicklow, Ireland).

### 2.2. Methods

#### 2.2.1. Bazlama Production and Quality Evaluation

Bazlama samples were prepared by supplementing wheat flour with barley flour at 15, 30, 45, and 60% supplementation levels. The flour obtained from a strong bread wheat cultivar (Tosunbey) was used to encounter the weakening effect of barley flour on bread-making quality. Bazlama samples were prepared according to the method specified by [20]. To prepare the bazlama sample, 200 g of flour (whole barley flour and refined wheat flour were mixed in certain proportions based on 14% moisture content), dry yeast (2%), sugar (1%), salt (1.5%), and water at 30 °C (Farinograph water absorption) were mixed at maximum speed using a dough mixer (Kitchen Aid, Classic 4.3 L, St. Joseph, MI, USA) until the dough developed. After mixing, the sample was left to ferment for 1 h at 30 °C. The dough samples were divided into 140 g pieces, given a round shape, kept at room temperature for 6 min, and then sheeted with a roller pin to a thickness of 9 mm. It was baked for 5 min at 240 ± 5 °C on an electrically heated pan (Goldmaster, 1200 W, İstanbul, Türkiye). The bazlama samples were turned upside down after 2.5 min of baking to have equal baking on both sides. After baking, the samples were cooled at 25 °C for 2 h and placed in plastic bags. Except for the texture analysis, the bazlama samples were cut into small pieces, dried at 40 °C, and ground using a grinder (Tefal, GT110838, Rumilly, France) for further analyses.

#### 2.2.2. Color Parameters of Bazlama Samples

The crumb and crust color values (L*, a*, b*) of the bazlama samples were measured using a colorimeter (Konica Minolta Sensing, Inc., CR-400, Osaka, Japonya) and expressed as L* (whiteness/darkness), a* (redness/greenness), and b* (yellowness/blueness) values. The total color difference (∆E) of bazlama samples was calculated as follows:

(1) $$\Delta E=\sqrt{{\left({\Delta L}^{*}\right)}^{2\;}+{\left({\Delta a}^{*}\right)}^{2}+{\left({\Delta b}^{*}\right)}^{2}}$$

#### 2.2.3. Texture Analysis of the Bazlama Samples

Texture profile analysis (TPA) of bazlama samples was performed using a TA-TX plus Texture Analyser (Stable Micro Systems, Surrey, UK), according to a developed method by Marchetti et al. [21]. The bazlama samples were divided into roughly four equal slices. For TPA, pre-test speed, post-test speed, and test speed were each arranged as 1.7 mm/s, and the compression was 30% of the height of the bazlama sample. An interval of 5 s between two compression cycles and a trigger force of 5 g were selected. A P/36 cylindrical probe (diameter: 36 mm; Stable Micro Systems, Surrey, UK) was used for TPA. TPA curves provided the parameters hardness, cohesiveness, chewiness, and springiness. The bazlama samples were tested after 2 h, 24 h, and 48 h of storage after baking.

#### 2.2.4. β-Glucan Content Determination of the Bazlama Samples

β-Glucan contents of the bazlama samples were evaluated by Megazyme β-glucan Assay Kit (Megazyme International, Wicklow, Ireland) in accordance with AACC International Method No. 32-23.01 and Method No. 32-40.01, respectively [22]. All of the analyses on the raw materials and bazlama samples were duplicated.

#### 2.2.5. Mineral Analysis of the Bazlama Samples by ICP-MS

For mineral analysis, the method of Kilic et al. [23] was followed using an inductively coupled plasma mass spectrometer (ICP-MS) (7700 Series x, Agilent, Wilmington, DE, USA) and a nebulizer (Agilent, Tokyo, Japan). The sample was digested by a microwave oven (1200 W, The Ethos up, Milestone Inc., Monroe, CT, USA) with a mixture of 6 mL of nitric acid (HNO_3_, 65%) and 2 mL of hydrogen peroxide (H_2_O_2_, 30%). The digested bazlama samples were evaporated to dryness and diluted to 20 mL with deionized water. Argon (99.95%) was the main, auxiliary, and nebulizer gas. The flow rates of the nebulizer and auxiliary gas were 0.70 and 0.20 L/min, respectively [23]. The results are given as μg/g.

#### 2.2.6. In Vitro Glycemic Index Value (GI) of the Bazlama Samples

The bazlama samples were digested according to the slightly modified version of the method reported by [24]. For this purpose, 10 glass beads (5 mm in diameter) were placed in a 50 mL tube, and then 100 mg of the sample was added into the tube. A total of 2 mL of 0.05 M HCl containing pepsin (5 mg/mL, Sigma, P7000, St. Louis, MO, USA) was added to the tubes. In a shaking water bath, the tubes were incubated at 37 °C for 30 min. Each tube was filled with 4 mL of 0.5 M sodium acetate buffer (pH 5.2), 1 mL of enzyme solution containing 0.104 g pancreatin (Sigma, P7545), and 14.45 U amyloglucosidase (3300 U/mL, Megazyme Int., Ireland), and the tubes were incubated at 37 °C for 90 min in a shaking water bath. Glucose content was determined with glucose oxidase–peroxidase reagent (Megazyme International, Wicklow, Ireland). The in vitro GI was calculated by using the following equation (Equation (2)) of [25].

*GI* = *39.71* + (*0.549* × *HI*) (2)

#### 2.2.7. Extraction and Determination of Free and Bound Phenolics from the Bazlama Samples

Bazlama samples were dried in a freeze dryer (Martin Christ, Beta 1–8 LSC plus, Osterode am Harz, Germany) for 48. Dried bazlama samples were ground and sieved through a 15-mesh sieve. Before analysis, the ground samples were defatted (three times) using hexane at a 1:5 (*w*/*v*) ratio. The samples were centrifuged at 2500× *g* for 5 min (Heraeus, Multifuge X3 FR, Thermo Scientific, Dreieich, Germany) after being shaken at 200 rpm for 10 min (MK200D, Yamato Scientific Co., Ltd., Tokyo, Japan). The samples were dried in a fume hood for 12 h [26,27]. Free and bound phenolic compounds of bazlama samples were extracted according to the method previously described by Shamanin et al. [27]. The free and bound phenolic contents were determined using the Folin–Ciocalteau reagent [28]. The total phenolic content (TPC) was calculated from the sum of free and bound phenolic compounds. The results are reported as mg gallic acid equivalent (GAE)/100 g dry weight (dw). 

#### 2.2.8. Determination of Antioxidant Capacities (DPPH and ABTS Methods) of the Bazlama Samples

The antioxidant capacities were determined using DPPH and ABTS methods. In the DPPH radical scavenging activity method described by Singh et al. [29], the absorbance values of free and bound extracts were determined using a spectrophotometer at 517 nm (Shimadzu 150 UV-1800, Kyoto, Japan). In the ABTS scavenging capacity method described previously [30], the absorbance was measured at 734 nm using the same spectrophotometer. The total antioxidant capacities were calculated from the sum of free and bound antioxidant capacities. The results are given as mg Trolox equivalent (TE)/100 g dry weight (dw).

#### 2.2.9. Statistical Analysis

All data obtained from analyses were presented in tables as the mean standard deviation. The mean standard deviation was presented for all data acquired from analyses. A statistics software (SPSS, IBM version 20, Armonk, NY, USA) was used for the statistical analysis. To assess the significant differences (*p* < 0.05), one-way ANOVA was employed, and Tukey’s post hoc test was utilized for comparisons of the means.

## 3. Results and Discussion

### 3.1. Color Properties of the Bazlama Samples 

The color properties of the bazlama samples are shown in Table 1. While the crumb color values (L*, a*, and b*) of the bazlama samples varied between 64.03 and 74.36, −0.46 and 3.61, and 17.98 and 24.07, respectively, the crust color values (L*, a*, and b*) of the bazlama samples varied between 65.10 and 73.36, −0.22 and 2.42, and 15.73 and 21.49, respectively. As the barley flour supplementation level increased, the L* values of the bazlama samples decreased (for both crumb and crust), indicating that Chifaa barley flour had a darkening effect on bazlama color. The difference between the crumb L* values of the control sample prepared with commercial wheat flour and the control sample prepared with Tosunbey wheat flour was not significant. All of the barley flour-enriched bazlama samples had higher yellowness values (b*) than the Tosunbey control bazlama sample (for both crumb and crust). This is a characteristic generally preferred by the consumers. The lower ΔE values mean that the barley flour-enriched bazlama samples have more resemblance with the control samples in terms of color. The color difference (ΔE) of crumb and crust ranged from 4.98 to 12.34 and 9.46 to 12.19, respectively. Both 15% and 45% of barley flour-enriched bazlama samples had similar ∆E values (both crumb and crust) and were not significantly different.

The composition and qualitative attributes of Turkish flatbread (bazlama) samples enriched with barley flour and wheat bran were studied [20]. The addition of barley flour reduced the *L* values of bazlama samples produced from two different bread wheat cultivars (Gerek and Gun), indicating a significant (*p* < 0.05) increase in greyish color. A similar trend was observed in the bazlama samples prepared with Chifaa barley flour and Tosunbey wheat flour in the present study. Despite the small changes noticed in the *a* and *b* values, there was no significant difference between the addition levels [20].

### 3.2. Textural Properties of the Bazlama Samples

Although the nutritional value of bread may be increased by the addition of β-glucan, incorporating β-glucan at the level recommended by various authorities (i.e., 3 g per day/0.75 g per serving) has proven to be difficult, leading to lower product quality and decreased consumer acceptability, especially in loaf-type breads. When preparing bread, substituting a significant percentage of wheat flour with non-gluten-forming flour such as barley flour will severely limit the viscoelasticity of the dough and the ability of blended dough matrices to retain gas. Weakened gluten networks frequently result in bread with reduced volume, texture, appearance, color, and sensory quality. The reduced gas holding capacity and lower volumes might be a major quality problem in loaf-type breads. However, these can be tolerated by flatbreads and do not pose a major quality problem since flatbreads have different quality evaluation criteria than those of loaf-type breads. A similar situation also applies to texture analysis results of different flatbreads.

Table 2 shows the hardness, cohesiveness, springiness, chewiness, and resilience characteristics of the bazlama samples. The force required to crush food between the teeth or between the tongue and the mouth to create deformation is defined as hardness.

The hardness of the bazlama samples increased significantly with the addition of 45 and 60% barley flour (*p* ˂ 0.05). Hardness was particularly increased above 45% barley inclusion. This would imply that the amount of barley flour addition should not exceed 45% in order to obtain a bazlama (flatbread) with acceptable quality. Mansoor et al. [31] also reported that the hardness of chickpea flour-based flatbread increased with added barley flour (10–40%). The resilience values of the barley flour-enriched bazlama samples were not significantly different compared to the control bazlama samples. The control (100% Tosunbey and commercial flour) bazlama samples had the highest cohesiveness properties. An increased barley flour supplementation in the bazlama formulations led to a significant decrease (*p* ˂ 0.05) in cohesiveness. The cohesiveness values of the control (100% Tosunbey: 0.89; commercial flour: 0.93) bazlama samples decreased to 0.87 and 0.86, respectively, after 48 h of storage. The cohesiveness of all bazlama samples decreased over the storage period (*p* ˂ 0.05). A similar observation of a decrease in the cohesiveness value and an increase in the hardness value of bread was reported [32]. According to Majzoobi et al. [33], as the percentage of wheat bran (5–20%) in the flatbreads raised, so did their hardness and cohesiveness values. The chewiness of all bazlama samples increased progressively during the storage of bread for up to 48 h at room temperature. Similar to the present study, El-Taib et al. [34] reported that the chewiness of all bread samples (wheat and 10–30% barley) increased gradually during storage of bread for up to 72 h at room temperature.

### 3.3. Estimated GI and HI Values and β-Glucan Contents of the Bazlama Samples

Kumar et al. [35] stated that, based on their GI values, foods can be classified as low (GI ≤ 55)-, medium (GI 56–69)-, and high (GI ≥ 70)-glycemic-index foods. The GI and HI values of the bazlama samples are presented in Table 3. There were significant differences between glycemic index values of the bazlama samples (*p* ˂ 0.05). The in vitro GI analysis results indicated that as the barley flour supplementation level increased, the in vitro GI values of the bazlama samples decreased significantly (*p* ˂ 0.05). Another parameter is the hydrolysis index (HI), which is calculated by dividing the area under the hydrolysis curve of each sample by the corresponding area of a reference sample (white bread) during the same period [36]. The HI of bazlama samples with all barley flour supplementation levels exhibited a significant decrease as compared to the control bazlama samples (commercial flour and 100% Tosunbey flour). The in vitro GI values decreased with the addition of barley flour to Tosunbey flour (Table 3). The GI-lowering effect of barley flour might transform bazlama from a high-GI food product to a medium-GI food product (for 45% and 60% barley flour-enriched bazlama samples). Mansoor et al. [31,37] also reported similar results when barley was added to chickpea- and wheat flour-based flatbread, respectively. Finocchiaro et al. [38] showed that the effectiveness of bread enriched with barley β-glucans in reducing GI was affected by the amylose/amylopectin ratio of the barley used. In a study by [39], glycemic index values of different types of bread were calculated as follows: bazlama (108.5), gluten-free bread (103.3), germ-enriched bread (84.1), ciabatta (83.5), whole wheat bread (77.6), and bread produced from stone mill flour (68.8), respectively.

The β-glucan contents of the bazlama samples enriched with different levels of barley flour and those of control samples are presented in Table 3. The β-glucan contents of the control samples prepared with 100% commercial flour and 100% Tosunbey flour were determined as 0.19 and 0.49 g/100 g on a dry basis, respectively. β-glucan contents of the bazlama samples enriched with barley flours significantly increased and were in the range of 0.79–2.83 g/100 g on a dry basis for the barley flour supplementation levels of 15 to 60%. In Turkey and various other Mediterranean countries, bread has been consumed in relatively higher amounts, and the consumption is generally in the range of 200–250 g/day [40,41]. According to bread consumption levels in Mediterranean countries, it could be estimated that the bazlama samples with 45% and 60% barley flour supplementation levels can provide around 2.5–3.0 g and 3.0–3.5 g of β-glucan, respectively, when 200–250 g of bread is consumed per day. Based on these results, 45% and 60% barley flour-enriched breads meet the requirements to bear the health claim by providing the required amount of β-glucan per day (3 g) with 200–250 g of bazlama consumption. 

### 3.4. Mineral Contents of the Bazlama Samples

The mineral composition of the bazlama samples is given in Table 4, and the contribution percentage of 200 g breads to mineral intake recommendation is given in Table 5. Barley was reported to have higher levels of Fe, Zn, Mn, Se, and Cu minerals compared to bread wheat [42]. According to the results of the present study, bazlama samples enriched with barley flour were found to have higher Mg, K, Mn, Fe, and Zn contents than the control sample, significantly (*p* < 0.05). Mg content increments of the bazlama samples were proportional to the barley flour supplementation level. Mg content increased by 84% in bazlama with 30% barley and 130% in bazlama with 60% barley compared to the control sample. A total of 200 g of bazlama with 60% barley is estimated to meet around 23% of the daily Mg requirement of a healthy female adult, whereas bazlama made from bread wheat flour is estimated to provide 9.8% of the daily Mg requirement based on intake recommendations [43]. The K contents of bazlama with 30% barley, bazlama with 45% barley, and bazlama with 60% barley samples were significantly higher than those of the control sample. K content increased by 47% in bazlama with 30% barley and 102% in bazlama with 60% barley compared to the control sample. A total of 200 g of barley flour-enriched bazlama is estimated to meet around 12% of the daily K requirement of a healthy adult, whereas bazlama made from bread wheat flour is estimated to provide 6% of the daily K requirement based on intake recommendations [43]. The Mn contents of bazlama with 30% barley, bazlama with 45% barley, and bazlama with 60% barley samples were significantly higher than that of the control sample. Mn content increased by 43% in bazlama with 30% barley and 67% in bazlama with 60% barley compared to the control sample. A total of 200 g of barley flour-enriched bazlama can meet 103% of the daily Mn requirement of a healthy female adult, whereas bazlama produced using only bread wheat provides 62% of the requirement based on intake recommendations [44]. The Fe contents of bazlama with 30% barley, bazlama with 45% barley, and bazlama with 60% barley samples were significantly higher than those of the control sample. Fe content increased by 103% in bazlama with 30% barley and by 293% in bazlama with 60% barley compared to the control sample. A total of 200 g of bazlama with 60% barley is estimated to meet around 82% of the daily Fe requirement of a healthy female adult, whereas bazlama made from bread wheat flour’s estimation is 21% [43]. Cu contents of all barley flour-enriched bazlama samples were significantly higher than that of the control sample (*p* < 0.05). Zn content increased by 67% in bazlama with 60% barley compared to the control sample.

### 3.5. Phenolic Contents and Antioxidant Capacities of the Bazlama Samples

Phenolic compounds are important constituents for plant pigmentation, growth, and reproduction, contributing to antioxidant activity [45]. Phenolic contents and antioxidant capacity values were determined as free and bound forms and are reported in Table 6. The free phenolic contents (FPC) of the control (commercial and 100% Tosunbey) and the barley flour-enriched bazlama samples varied from 173.89 to 219.29 mg GAE/100 g dw. Ragaee et al. [46] reported that oat-enriched bread contained the highest level of free phenolics at the level of 168 µg/g, followed by rye-enriched (139 µ/g), wheat-enriched (121 µ/g), and barley-enriched (119 µ/g) bread, and these results are lower compared to the results of the present study. As expected, the majority of phenolics were in the bound form. Compared to the control bazlama samples (commercial and 100% Tosunbey flours), 60% barley flour-enriched bazlama had the highest amount of bound phenolics (229.57 mg/100 g dw), followed by 45% (224.45 mg/100 g dw) and 30% (206.19 mg/100 g) barley flour-enriched ones (Table 6). As the barley flour supplementation level of the bazlama samples increased, the free and bound phenolics increased compared to the 100% Tosunbey bazlama sample. It was previously reported that phenolics in cereal-based matrices are mainly in the bound form. In addition, bound phenolics are covalently bound to structural components of the cell wall [47].

The TPC of control (commercial and 100% Tosunbey) and barley flour-enriched bazlama samples varied from 357.43 to 448.94 mg GAE/100 g dw. Mushtaq et al. [48] reported similar findings. When *Moringa oliefera* leaf powders were added to the flatbread, the TPC increased significantly from 75 mg GAE/100 g to 482 mg GAE/100 g. Del Carmen Robles-Ramírez et al. [49] reported that the substitution with 60% barley flour increased the content of the TPC of bread by 41.5%. In another study, the TPC of the flatbread (wheat flour) was reported as 14.43 mg GAE/100 g DW; after 25% lotus root flour blending, the TPC of flatbread increased to 96.48 mg GAE/100 g DW [50].

Due to their complex structure, the antioxidant capacities of grain samples are generally assessed using more than one method. Hence, in the present study, two different methods were used for the estimation of antioxidant capacity. In this study, ABTS and DPPH methods were used for antioxidant capacity determination. Table 6 shows the DPPH values for free and bound phenolics, and the values varied from 21.95 to 27.76 mg TE/100 g dw (commercial and 100% Tosunbey bazlama) and 37.31 to 55.54 mg TE/100 g dw (15–60% barley flour-enriched bazlama samples) for free phenolics. The DPPH values ranged from 32.13 to 35.39 mg TE/100 g dw (commercial and 100% Tosunbey bazlama) and 40.52 to 64.31 mg TE/100 g dw (15–60% barley flour-enriched bazlama samples) for bound phenolics. Similarly, the controls (commercial and 100% Tosunbey) showed the lowest total DPPH values (54.08 and 63.15 mg TE/100 g dw). The results of the ABTS and DPPH analyses showed similarities; as expected, the ABTS values of the control (commercial and 100% Tosunbey) samples were lower than the barley flour-enriched bazlama samples. Holtekjolen et al. [51] also stated that the addition of 40 g/100 g of barley in wheat baking formulas increased the antioxidant capacities of the breads compared to the control. In the present study, the total ABTS values of barley flour-enriched bazlama samples varied from 208.08 to 790.43 mg TE/100 g dw, while in a study by del Carmen Robles-Ramírez et al. [49], the total ABTS values of 100% wheat flour and 60% barley flour bread was found to be 1.71 and 2.63 µmol Trolox/g dw, respectively. Compared to the present study, these ABTS values are relatively lower. Saeed et al. [50] reported that flatbread prepared from lotus root flour–wheat flour blends showed higher radical scavenging activity (DPPH value) compared to the control (wheat flour flatbread). The DPPH and ABTS values of barley flour-enriched bazlama samples demonstrated the utilization possibility of this cereal to increase the antioxidant capacities of bazlama.

## 4. Conclusions

Barley is a significant staple food in the Middle East and North African countries and has recently started to recuperate importance in many countries. The goal of this project (MEDWHEALTH) is the re-designing of some of the major Med foods to increase their health benefits by utilizing novel raw materials such as high-β-glucan barley. Traditional flatbreads such as bazlama are commonly consumed in various Mediterranean countries. Hence, improving the healthy ingredients of bazlama will be an advantage for the people consuming such products. The present study indicated that supplementing wheat flour with barley flour resulted in bazlama samples with improved nutritional properties. The contents of β-glucan, phenolics, and minerals, as well as the antioxidant capacity of the bazlama samples, increased considerably. Thus, barley-enriched bazlama samples (45 and 60%) have the potential to be recognized as a functional food with better antioxidant capacity. The results of the present study indicated that high-β-glucan barley can be utilized to produce bazlama with higher nutritional properties. Furthermore, the findings of this study may provide insights that can contribute to future studies in the domain of functional foods. Since bread is regarded as a staple food in many countries around the world, the availability of various composite wheat–barley flour flatbreads on the market would likely contribute to an increase in the consumption of barley flour in the future.

## Figures and Tables

**Table 1:** Color values of the bazlama samples.

	Crumb Color				Crust Color			
Sample	L*	a*	b*	ΔE	L*	a*	b*	ΔE
Control (Commercial)	74.36 ± 0.82 ^a^	−0.36 ± 0.23 ^d^	17.98 ± 0.43 ^c^		67.93 ± 0.63 ^bc^	−0.18 ± 0.02 ^d^	18.88 ± 0.82 ^ab^	
Control (100% Tosunbey)	73.88 ± 0.79 ^a^	−0.46 ± 0.04 ^d^	19.36 ± 0.07 ^bc^		73.36 ± 0.30 ^a^	−0.22 ± 0.04 ^d^	15.73 ± 0.04 ^b^	
Bazlama (15% Barley)	71.79 ± 1.02 ^ab^	0.93 ± 0.02 ^c^	22.12 ± 0.40 ^ab^	4.98 ± 0.78 ^c^	71.10 ± 1.43 ^ab^	0.75 ± 0.04 ^c^	18.91 ± 0.62 ^ab^	9.46 ± 1.01 ^b^
Bazlama (30% Barley)	68.84 ± 0.20 ^bc^	1.49 ± 0.06 ^bc^	19.64 ± 0.39 ^bc^	5.70 ± 0.67 ^c^	70.80 ± 0.58 ^ab^	1.14 ± 0.18 ^c^	19.43 ± 0.87 ^a^	9.31 ± 1.00 ^b^
Bazlama (45% Barley)	67.36 ± 0.54 ^cd^	2.26 ± 0.18 ^b^	22.31 ± 0.90 ^ab^	8.39 ± 0.42 ^b^	68.44 ± 0.63 ^bc^	1.77 ± 0.01 ^b^	21.04 ± 0.41 ^a^	10.43 ± 0.90 ^ab^
Bazlama (60% Barley)	64.03 ± 0.09 ^d^	3.61 ± 0.23 ^a^	24.07 ± 1.22 ^a^	12.34 ± 0.87 ^a^	65.10 ± 0.29 ^c^	2.42 ± 0.05 ^a^	21.49 ± 0.48 ^a^	12.19 ± 0.80 ^a^

^a–d^ Means with different letters within each column are significantly different (*p* < 0.05).

**Table 2:** Textural properties of the bazlama samples.

Sample	Hardness (N)	Springiness	Cohesiveness	Gumminess	Chewiness	Resilience
2 h						
Control (Commercial)	4.92 ± 0.56 ^c^	0.98 ± 0.02 ^a^	0.93 ± 0.01 ^a^	3.99 ± 0.86 ^bc^	3.87 ± 0.737 ^bc^	0.56 ± 0.01 ^ab^
Control (100% Tosunbey)	4.64 ± 0.69 ^c^	0.97 ± 0.01 ^a^	0.89 ± 0.01 ^ab^	4.14 ± 0.65 ^bc^	4.03 ± 0.635 ^bc^	0.52 ± 0.02 ^ab^
Bazlama (15% Barley)	3.42 ± 0.06 ^c^	0.95 ± 0.01 ^a^	0.89 ± 0.01 ^ab^	3.83 ± 0.82 ^bc^	3.62 ± 0.77 ^bc^	0.50 ± 0.03 ^ab^
Bazlama (30% Barley)	4.78 ± 0.38 ^c^	0.94 ± 0.02 ^a^	0.89 ± 0.01 ^ab^	2.32 ± 0.55 ^c^	2.18 ± 0.51 ^c^	0.45 ± 0.04 ^b^
Bazlama (45% Barley)	11.28 ± 0.22 ^b^	0.96 ± 0.04 ^a^	0.88 ± 0.01 ^ab^	8.43 ± 0.91 ^b^	8.14 ± 0.88 ^b^	0.57 ± 0.01 ^ab^
Bazlama (60% Barley)	32.65 ± 0.71 ^a^	0.96 ± 0.01 ^a^	0.87 ± 0.01 ^b^	28.83 ± 0.95 ^a^	27.62 ± 0.88 ^a^	0.58 ± 0.01 ^a^
24 h						
Control (Commercial)	10.41 ± 0.21 ^c^	1.58 ± 0.11 ^a^	0.89 ± 0.02 ^a^	8.88 ± 0.09 ^bc^	12.30 ± 0.83 ^bc^	0.60 ± 0.01 ^a^
Control (100% Tosunbey)	8.23 ± 0.06 ^c^	0.97 ± 0.00 ^b^	0.88 ± 0.01 ^a^	14.38 ± 0.71 ^bc^	14.73 ± 1.47 ^bc^	0.57 ± 0.02 ^a^
Bazlama (15% Barley)	7.63 ± 0.21 ^c^	0.97 ± 0.01 ^b^	0.88 ± 0.01 ^a^	9.22 ± 0.41 ^bc^	8.91 ± 0.38 ^bc^	0.62 ± 0.01 ^a^
Bazlama (30% Barley)	10.53 ± 0.46 ^c^	0.97 ± 0.01 ^b^	0.87 ± 0.01 ^a^	4.94 ± 1.92 ^c^	4.78 ± 1.80 ^c^	0.58 ± 0.03 ^a^
Bazlama (45% Barley)	25.18 ± 0.05 ^b^	0.98 ± 0.01 ^b^	0.87 ± 0.01 ^a^	26.29 ± 4.50 ^ab^	25.43 ± 4.45 ^ab^	0.60 ± 0.01 ^a^
Bazlama (60% Barley)	44.48 ± 3.72 ^a^	0.97 ± 0.02 ^b^	0.87 ± 0.01 ^a^	43.23 ± 6.95 ^a^	41.82 ± 5.62 ^a^	0.63 ± 0.01 ^a^
48 h						
Control (Commercial)	15.80 ± 0.71 ^d^	0.98 ± 0.02 ^a^	0.87 ± 0.02 ^a^	13.26 ± 0.81 ^b^	13.51 ± 0.54 ^b^	0.60 ± 0.03 ^a^
Control (100% Tosunbey)	26.48 ± 1.34 ^c^	1.16 ± 0.05 ^a^	0.86 ± 0.01 ^a^	20.34 ± 3.42 ^b^	23.80 ± 4.93 ^b^	0.57 ± 0.02 ^a^
Bazlama (15% Barley)	15.86 ± 0.19 ^d^	0.97 ± 0.01 ^a^	0.85 ± 0.01 ^ab^	19.89 ± 0.62 ^b^	14.44 ± 1.32 ^b^	0.58 ± 0.01 ^a^
Bazlama (30% Barley)	23.22 ± 1.10 ^c^	0.95 ± 0.01 ^a^	0.85 ± 0.01 ^ab^	15.18 ± 1.47 ^b^	19.33 ± 0.45 ^b^	0.56 ± 0.01 ^a^
Bazlama (45% Barley)	48.52 ± 0.07 ^b^	1.31 ± 0.32 ^a^	0.84 ± 0.01 ^b^	46.07 ± 1.78 ^a^	44.53 ± 2.42 ^a^	0.62 ± 0.01 ^a^
Bazlama (60% Barley)	56.58 ± 0.39 ^a^	0.95 ± 0.01 ^a^	0.84 ± 0.05 ^b^	50.10 ± 2.18 ^a^	47.72 ± 2.23 ^a^	0.61 ± 0.04 ^a^

^a–d^ Means with different letters within each column are significantly different (*p* < 0.05).

**Table 3:** Hydrolysis index and in vitro GI values and β-glucan contents of the bazlama samples.

Samples	HI	GI	β-Glucan (%)
Control (Commercial)	88.60 ± 1.44 ^a^	92.10 ± 0.80 ^a^	0.19 ± 0.01 ^f^
Control (100% Tosunbey)	79.20 ± 0.55 ^b^	83.19 ± 0.30 ^b^	0.49 ± 0.01 ^e^
Bazlama (15% Barley)	71.25 ± 0.78 ^c^	78.83 ± 0.43 ^c^	0.79 ± 0.01 ^d^
Bazlama (30% Barley)	63.93 ± 1.11 ^d^	74.81 ± 0.61 ^d^	1.45 ± 0.06 ^c^
Bazlama (45% Barley)	52.72 ± 0.73 ^e^	68.65 ± 0.40 ^e^	1.86 ± 0.01 ^b^
Bazlama (60% Barley)	45.57 ± 0.60 ^f^	64.73 ± 0.33 ^f^	2.83 ± 0.01 ^a^

^a–f^ Means with different letters within each column are significantly different (*p* < 0.05). HI: hydrolysis index; GI: glycemic index.

**Table 4:** Mineral contents of the bazlama samples.

	Mg	K	Ca	Mn	Fe	Cu	Zn
Control (100% Tosunbey)	236.2 ± 14.4 ^c^	2120 ± 130 ^c^	66.04 ± 1.84 ^b^	8.55 ± 0.28 ^c^	28.70 ± 0.95 ^d^	1.68 ± 0.05 ^c^	6.23 ± 0.18 ^c^
Bazlama (15% Barley)	297.3 ± 18.2 ^c^	2400 ± 150 ^c^	71.40 ± 1.99 ^ab^	9.56 ± 0.32 ^c^	33.01 ± 1.10 ^d^	1.88 ± 0.05 ^b^	9.20 ± 0.25 ^b^
Bazlama (30% Barley)	435.4 ± 26.6 ^b^	3120 ± 190 ^b^	75.33 ± 2.09 ^a^	12.18 ± 0.41 ^b^	58.30 ± 1.94 ^c^	1.97 ± 0.06 ^b^	9.38 ± 0.27 ^b^
Bazlama (45% Barley)	480.2 ± 29.3 ^ab^	3770 ± 230 ^a^	74.08 ± 2.00 ^a^	13.07 ± 0.42 ^b^	67.20 ± 2.24 ^b^	2.37 ± 0.07 ^a^	9.50 ± 0.27 ^b^
Bazlama (60% Barley)	542.5 ± 33.1 ^a^	4300 ± 260 ^a^	78.21 ± 2.12 ^a^	14.31 ± 0.46 ^a^	113.00 ± 3.90 ^a^	2.23 ± 0.06 ^a^	10.38 ± 0.29 ^a^

^a–d^ Means with different letters within each row are significantly different (*p* < 0.05). Control: 100% Tosunbey flour. Mineral value of samples is expressed as μg/g.

**Table 5:** Contribution of 200 g of bazlama bread to mineral intake recommendation.

	Mg (mg/d)	K (g/d)	Mn (mg/d)	Fe (mg/d)	Zn (mg/d)
Intake Recommendation	*F: 310 *M: 400	F: 4.7 M: 4.7	F: 1.8 M: 2.3	F: 18 M: 8	F: 8 M: 11
Breads	Percentage of 200 g of bazlama bread meeting daily mineral requirement (%)				
Control (100% Tosunbey)	F: 9.8 M: 7.6	F: 6 M: 6	F: 62 M: 48	F: 21 M: 47	F: 10 M: 7
Bazlama (15% Barley)	F: 12 M: 10	F: 7 M: 7	F: 69 M: 54	F: 24 M: 54	F: 15 M: 11
Bazlama (30% Barley)	F: 18 M: 14	F: 9 M: 9	F: 88 M: 68	F: 42 M: 95	F: 15 M: 11
Bazlama (45% Barley)	F: 20 M: 16	F: 10 M: 10	F: 94 M: 74	F: 49 M: 109	F: 15 M: 11
Bazlama (60% Barley)	F: 23 M: 18	F: 12 M: 12	F: 103 M: 81	F: 82 M: 180	F: 17 M: 12

Control: 100% Tosunbey flour. * F and M: healthy adults between 19 and 30 years of age, female and male.

**Table 6:** Phenolic content and antioxidant capacities (ABTS and DPPH methods) of the bazlama samples.

Free Fraction			
Sample	Phenolic Content	DPPH	ABTS
Control (Commercial)	173.89 ± 0.33 ^e^	21.95 ± 0.39 ^e^	26.59 ± 0.35 ^e^
Control (100% Tosunbey)	176.07 ± 0.98 ^e^	27.76 ± 0.39 ^d^	34.95 ± 0.35 ^e^
Bazlama (15% Barley)	184.19 ± 1.32 ^d^	37.31 ± 1.17 ^c^	105.71 ± 3.15 ^d^
Bazlama (30% Barley)	201.17 ± 0.34 ^c^	44.20 ± 1.59 ^b^	122.16 ± 0.72 ^c^
Bazlama (45% Barley)	213.32 ± 1.31 ^b^	48.86 ± 2.32 ^b^	250.65 ± 5.22 ^b^
Bazlama (60% Barley)	219.29 ± 0.98 ^a^	55.54 ± 0.77 ^a^	390.33 ± 5.18 ^a^
**Bound Fraction**			
Control (Commercial)	183.55 ± 0.46 ^d^	32.13 ± 1.56 ^e^	76.61 ± 1.05 ^f^
Control (100% Tosunbey)	185.32 ± 0.33 ^d^	35.39 ± 0.39 ^e^	89.56 ± 2.60 ^e^
Bazlama (15% Barley)	191.65 ± 0.23 ^c^	40.52 ± 1.72 ^d^	102.37 ± 1.22 ^d^
Bazlama (30% Barley)	206.19 ± 2.71 ^c^	48.89 ± 1.39 ^c^	123.43 ± 0.72 ^c^
Bazlama (45% Barley)	224.45 ± 4.59 ^b^	58.43 ± 1.48 ^b^	294.93 ± 1.74 ^b^
Bazlama (60% Barley)	229.57 ± 1.30 ^a^	64.31 ± 1.97 ^a^	400.10 ± 1.73 ^a^
* Total			
Control (Commercial)	357.43 ± 0.13 ^f^	54.08 ± 1.17 ^e^	103.20 ± 0.70 ^f^
Control (100% Tosunbey)	361.39 ± 1.31 ^e^	63.15 ± 0.03 ^e^	124.51 ± 2.26 ^e^
Bazlama (15% Barley)	375.83 ± 1.32 ^d^	77.83 ± 2.89 ^d^	208.08 ± 1.92 ^d^
Bazlama (30% Barley)	407.35 ± 3.04 ^c^	93.09 ± 2.99 ^c^	245.58 ± 1.44 ^c^
Bazlama (45% Barley)	437.77 ± 3.28 ^b^	107.29 ± 3.80 ^b^	545.59 ± 3.48 ^b^
Bazlama (60% Barley)	448.94 ± 0.33 ^a^	119.85 ± 2.74 ^a^	790.43 ± 3.45 ^a^

^a–f^ Means with different letters in the same column are significantly different (*p* < 0.05). Phenolic contents are expressed as mg GAE/100 g dry weight (dw). ABTS: 2,2′-azino-bis (3-ethyl-benzothiazoline6-sulphonic acid); DPPH: 2,2-diphenyl-1-picrylhydrazyl radical scavenging activity. * The sum of free and bound antioxidant capacities expressed as mg TE/100 g dw.

## Data Availability

The data is contained in the article.
